# Antiviral Treatment for Congenital Cytomegalovirus Infection in Extremely Preterm Newborn: A Case Report and Literature Review

**DOI:** 10.3390/v18030391

**Published:** 2026-03-20

**Authors:** Giovanni Boscarino, Giusy Davino, Silvia Pezzoni, Mara Corradi, Maria Carmela Pera, Susanna Esposito, Enzo Romanini

**Affiliations:** 1 Pediatric Clinic, Pietro Barilla Children’s Hospital, University Hospital of Parma, 43126 Parma, Italy; giovanni.boscarino@unipr.it (G.B.); mariacarmela.pera@unipr.it (M.C.P.); 2 Department of Medicine and Surgery, University of Parma, 43126 Parma, Italy; 3 Neonatal Intensive Care Unit, Pietro Barilla Children’s Hospital, University Hospital of Parma, 43126 Parma, Italyeromanini@ao.pr.it (E.R.)

**Keywords:** CMV, ganciclovir, valganciclovir, neutropenia, prematurity, antiviral, critical ill newborn

## Abstract

Background: Congenital cytomegalovirus (cCMV) infection is one of the most common congenital infections worldwide and the leading cause of non-genetic sensorineural hearing loss. Although less frequent in preterm infants, cCMV may significantly worsen outcomes in an already vulnerable population. The risks and benefits of antiviral therapy in extremely preterm neonates remain unclear, as this group is largely excluded from clinical trials. Case presentation: We report a case of symptomatic cCMV infection in an extremely preterm infant born at 26 weeks and 2 days of gestation to a mother with primary CMV infection during the second trimester. High CMV viral loads were detected in urine and plasma shortly after birth. On day of life (DOL) 3, respiratory deterioration required intubation, with radiological findings consistent with CMV pneumonia and positive bronchoaspirate samples. Intravenous ganciclovir was initiated on DOL 16 and administered for six weeks, followed by oral valganciclovir for six months. Treatment was associated with a favourable clinical and virological response and no significant hematological toxicity. Ophthalmologic and audiological evaluations were normal. Neurodevelopmental assessment with Bayley III at one year of corrected age demonstrated age-appropriate performance across all domains. Discussion: A structured literature review identified 10 case reports, including 13 extremely preterm infants treated for cCMV infection. Antiviral dosing regimens were heterogeneous. The most frequent manifestations prompting treatment were laboratory abnormalities (92.3%), particularly thrombocytopenia and leukopenia or neutropenia. Neuroimaging abnormalities and intrauterine growth restriction or small for gestational age were each reported in 53.8% of cases. Long-term neurodevelopmental outcomes were normal in 38.5% of infants. Conclusions: Antiviral therapy for cCMV infection with ganciclovir and valgancyclovir in premature neonates is feasible and safe with careful monitoring, and appears to provide benefits. Nevertheless, well-designed studies that include pharmacokinetics and pharmacodynamics, virologic monitoring, and long term outcomes of development, vision and hearing are urgently needed.

## 1. Introduction

Congenital cytomegalovirus (cCMV) infection is among the most common congenital infections worldwide and represents the leading cause of non-genetic sensorineural hearing loss [1]. Although the majority of infected neonates are asymptomatic at birth, approximately 10–15% present with clinical manifestations [2]. Importantly, accumulating evidence indicates that even infants with normal audiological screening at birth remain at risk for late-onset hearing loss, which may adversely affect long-term neurodevelopmental outcomes [3]. The clinical spectrum of cCMV is broad, and considerable heterogeneity persists in the definition of “symptomatic” infection, leading to variability in treatment indications and management strategies across different healthcare settings [4].

The incidence of cCMV infection in preterm infants is generally lower than in term neonates because the risk of transmission increases across gestation, with a higher risk of transmission during the third trimester [5]. In this population, CMV infection is more commonly acquired postnatally, predominantly through breast milk from seropositive mothers [6]. Nevertheless, congenital infection does occur in preterm infants, with reported incidence rates ranging from 0.4% to 2% [7,8]. When present, cCMV infection may substantially worsen outcomes in an already fragile population characterized by marked physiological and immunological immaturity, increasing susceptibility to severe and disseminated disease, including pneumonitis, hepatitis, colitis, and thrombocytopenia [8,9,10]. Despite these risks, current clinical guidelines do not specifically address the management of extremely preterm infants and fail to provide clear treatment recommendations for neonates born before 32 weeks of gestational age (GA) or with extremely low birth weight (ELBW) [11].

Antiviral therapy with ganciclovir (GC) or its oral prodrug valganciclovir (VGC) has been associated with improved outcomes in symptomatic cCMV infection but is also linked to potentially serious adverse effects, particularly neutropenia and thrombocytopenia [11,12]. These complications may further compromise clinical stability in extremely preterm infants and complicate the assessment of the risk–benefit balance of treatment. To date, no randomized clinical trials have specifically evaluated antiviral therapy in this high-risk population, as extremely preterm neonates are routinely excluded from most interventional studies.

Available evidence supports early initiation of antiviral therapy—ideally within the first month of life—as a key determinant of improved outcomes in severe cCMV infection [13]. However, the profound physiological and immunological differences between preterm and term neonates render this therapeutic window difficult to interpret in extremely preterm infants, for whom the first month of postnatal life does not correspond to the same developmental stage as in term newborns [14].

Furthermore, optimal GC dosing and treatment duration for preterm infants with end-organ CMV disease remain poorly defined [15]. While studies in term neonates support an initial GC dosage of 6 mg/kg every 12 h [11], pharmacokinetic data in preterm infants are limited. A recent study by Acosta et al. suggested that a lower initial dose of 5 mg/kg every 12 h may be appropriate in preterm neonates with cCMV infection [16]. However, robust data are lacking, and further well-conducted randomized clinical trials are needed to establish evidence-based dosing strategies and to better delineate the balance between efficacy and safety in extremely preterm infants.

In this context, we report a case of symptomatic cCMV infection in an extremely preterm neonate successfully treated with antiviral therapy and present a systematic review of similar cases reported in the literature. To this end, we conducted a structured electronic search of MEDLINE, Scopus, and the Cochrane Library using predefined Medical Subject Headings (MeSH) and Boolean operators: congenital AND (cytomegalovirus OR CMV) AND (neonate OR newborn OR infant) AND (preterm OR premature) AND (antiviral OR treatment OR therapy OR valganciclovir OR ganciclovir). We included all English-language case reports published up to 31 December 2025, involving extremely preterm neonates (≤32 weeks’ gestation) treated for cCMV infection.

Two authors (G.B. and G.D.) independently screened eligible studies and extracted predefined variables using a structured data collection form. Extracted data included gestational age, birth weight, clinical presentation of cCMV infection, antiviral treatment regimens and associated complications, mortality, and long-term neurological outcomes. Due to the descriptive nature of the included studies, no formal statistical analyses or risk-of-bias assessments were performed.

## 2. Case Presentation

We describe the case of an extremely preterm male infant born at 26 weeks and 2 days of GA with an appropriate weight for GA. The neonate had an extremely low birth weight of 930 g (Z-score: 0.39) and a head circumference of 23.5 cm (Z-score: −0.56), with no evidence of microcephaly at birth. This was the mother’s first pregnancy. Delivery occurred spontaneously following suspected placental abruption, onset of labor, and premature rupture of membranes. Maternal vaginal–rectal screening was positive for Group B *Streptococcus*. During pregnancy, the mother experienced a primary CMV infection in the second trimester and, although the current indication is to treat women with primary infection during the first trimester of pregnancy, she received antiviral therapy with valacyclovir (8 g/day) starting at 21 weeks’ gestation. Antenatal corticosteroid prophylaxis with betamethasone was administered according to standard protocols. No additional infectious complications were reported during pregnancy.

At birth, the infant showed moderate respiratory effort and required intermittent positive pressure ventilation, with Apgar scores of 7 and 8 at 1 and 5 min, respectively, indicating a moderate adaptation to extrauterine life despite extreme prematurity. He was transferred to the neonatal intensive care unit on nasal continuous positive airway pressure with a fraction of inspired oxygen (FiO_2_) of 21%. Caffeine therapy was initiated, and empirical broad-spectrum antibiotics (ampicillin and gentamicin) were started due to the maternal infectious history while awaiting blood culture results. Antibiotic therapy was discontinued on day of life (DOL) 7 following negative culture findings.

Initial chest radiography was consistent with respiratory distress syndrome, and the infant received surfactant therapy (200 mg/kg) using the INtubation–SURfactant–Extubation technique. On DOL 3, respiratory status worsened, necessitating re-intubation and initiation of controlled volume-guaranteed mechanical ventilation, along with a second dose of surfactant (100 mg/kg). Chest radiography at that time demonstrated reticular–nodular opacities and parenchymal thickening in the right apical region. Creatinine and liver enzymes were in normal range values.

**Figure 1:**
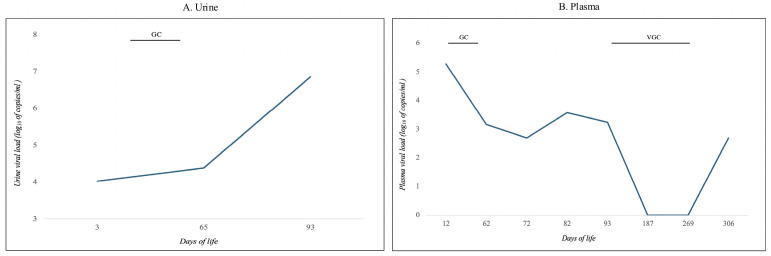
CMV-DNA urine and blood sample monitoring. (**A**) Urine samples; (**B**) Plasma samples.

Given the maternal history of primary CMV infection, urine testing for CMV DNA was performed on DOL 3 and revealed a viral load of 10,624 copies/mL (Figure 1A, log_10_ scale). Plasma CMV DNA measured on DOL 12 was 188,915 copies/mL (Figure 1B, log_10_ scale). These values might be related to a recent infection with a subsequent increase in the viral load related to the natural history of cCMV infection.

**Figure 2:**
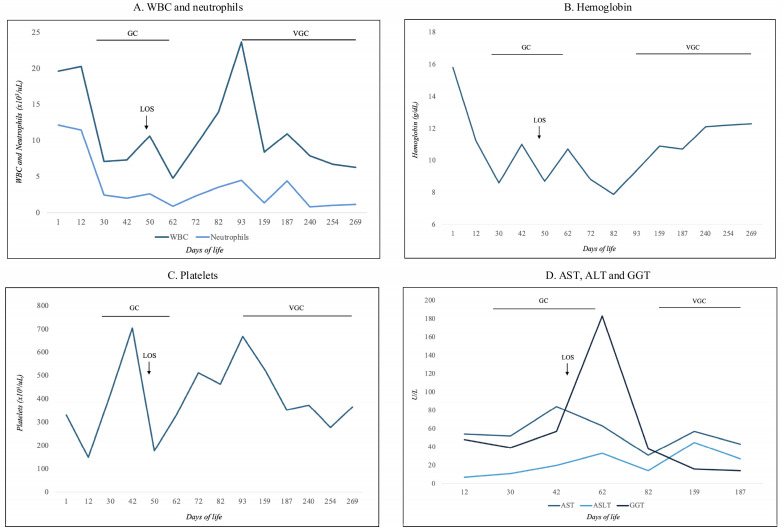
Blood sample monitoring. (**A**) White Blood Cell (WBC) and neutrophils; (**B**) Hemoglobin; (**C**) Platelts; (**D**) Aspartate Aminotransferase (AST), Alanine Aminotransferase (ALT) and GGT (Gamma-glutamyl Transferase).

As respiratory deterioration progressed, requiring invasive mechanical ventilation, microbiological analysis of bronchoaspirate samples identified CMV and *Candida albicans*. In the context of possible symptomatic cCMV infection with pulmonary involvement, antiviral therapy with intravenous GC (6 mg/kg twice daily) was initiated on DOL 16 (corrected GA 28 + 3 weeks) and continued for six weeks, with close laboratory monitoring (Figure 2).

Concomitant intravenous fluconazole was started at therapeutic dosage.

During hospitalization, CMV DNA levels were serially monitored in plasma and urine (Figure 1, log_10_ scale). Plasma viral load initially declined following initiation of GC but showed a subsequent increase toward the end of the treatment course. Approximately 17 days after starting GC, with progressive improvement in respiratory status, the infant was extubated and transitioned to non-invasive ventilation. Respiratory support was gradually weaned and discontinued at 36 + 3 weeks’ GA, and the infant was diagnosed with grade 2 bronchopulmonary dysplasia [17].

Following consultation with a pediatric infectious disease specialist, oral valganciclovir (VGC) therapy was initiated at 39 + 3 weeks’ GA (DOL 93) and continued for six months, with ongoing multidisciplinary follow-up. Plasma CMV DNA became undetectable at the end of VGC therapy and remained below 500 copies/mL one month after treatment discontinuation (Figure 1B).

On DOL 48, the infant developed late-onset sepsis, with blood cultures positive for *Klebsiella oxytoca*. This episode was associated with increased apnoeic events and elevated inflammatory markers (procalcitonin 51.62 ng/mL; C-reactive protein 63.2 mg/L). Empirical intravenous therapy with vancomycin and meropenem was initiated. Based on antibiotic susceptibility testing, vancomycin was discontinued after four days, while meropenem was continued for a total of 14 days until blood cultures cleared and clinical resolution was achieved.

Serial cranial ultrasounds demonstrated periventricular hyperechogenicity. Brain magnetic resonance imaging (MRI) performed at 38 + 5 weeks’ GA revealed symmetric periventricular white matter signal abnormalities, most pronounced in the peritrigonal regions. A follow-up brain MRI obtained at the end of VGC therapy, at 10 months of corrected age, showed complete resolution of these abnormalities.

Neurological evaluation by a child neuropsychiatrist identified mild prematurity-related findings, including initial hypotonia, which largely resolved during hospitalization. Poor visual tracking was noted early, although fixation was preserved. Serial ophthalmologic examinations revealed no evidence of retinopathy of prematurity or chorioretinal lesions attributable to cCMV during hospitalization and at 12 months of corrected age. Auditory evoked potentials performed at discharge and during follow-up (at 3, 6 and 12 months of corrected age) were normal.

During hospitalization, the infant experienced prematurity-related jaundice and required three red blood cell transfusions for anemia. Hematological and biochemical parameters—including white blood cell count, neutrophils, hemoglobin, platelets, aspartate aminotransferase, alanine aminotransferase, and gamma-glutamyl transferase—were monitored regularly throughout antiviral therapy (Figure 2). No abnormalities necessitated treatment interruption. The lowest absolute neutrophil count was 800/μL on DOL 240 during VGC therapy, with spontaneous recovery to 1010/μL approximately 15 days later (Figure 2A).

At approximately one year of corrected age, neurodevelopmental assessment using the Bayley Scales of Infant and Toddler Development, Third Edition (Bayley III), demonstrated normal development across all domains: Cognitive scale (scaled score 10; composite score 100), Language scale (scaled score 16; composite score 89), Motor scale (scaled score 19; composite score 97), and Socioemotional competence (scaled score 11; composite score 105).

## 3. Discussion

This case report supports the notion that antiviral therapy for cCMV infection in extremely preterm infants can be both effective and safe when carefully selected and closely monitored.

Based on our systematic review, the present case represents the 13th reported extremely preterm neonate treated with antiviral therapy for cCMV infection. Overall, reported complication rates remain limited, and neurological outcomes appear favourable in a subset of survivors.

**Figure 3:**
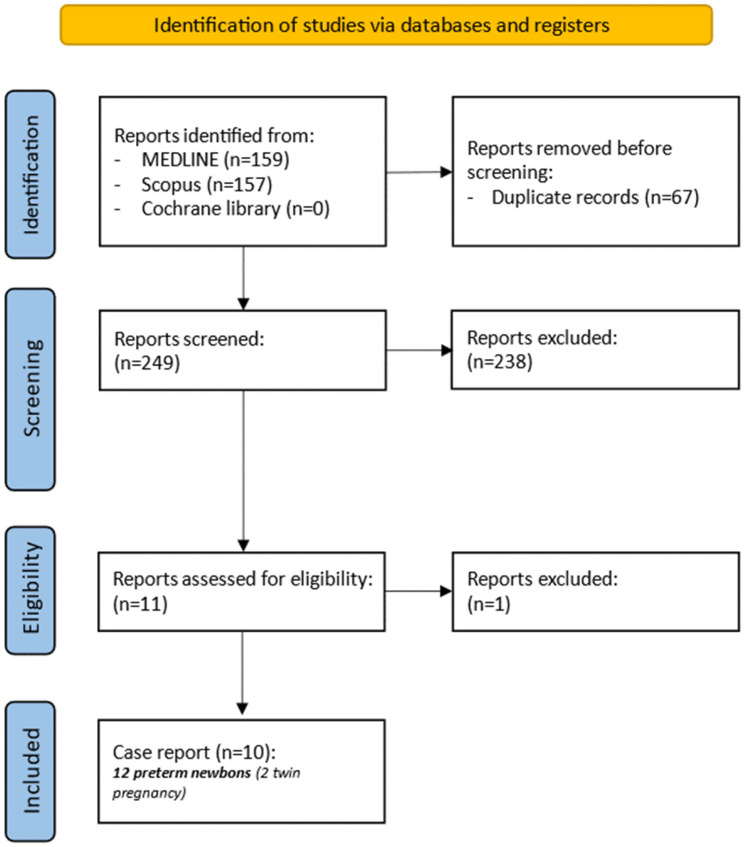
PRISMA flow-chart.

As illustrated in Figure 3, the initial search across databases and registers identified 316 records. After removal of 67 duplicates, 10 case reports met the predefined inclusion criteria and were included in the final analysis [18,19,20,21,22,23,24,25,26,27]. No additional studies were identified through alternative search strategies. Accounting for twin pregnancies, a total of 12 extremely preterm neonates were identified in the literature; when combined with our case, this resulted in a cohort of 13 extremely preterm infants treated for cCMV infection.

Table 1 summarizes neonatal characteristics, antiviral regimens, treatment-related complications, mortality, and long-term neurodevelopmental outcomes.

Considerable heterogeneity was observed in antiviral dosing strategies, particularly for GC. Among the 13 infants, three died. Of the remaining 10 survivors, five discontinued antiviral therapy prematurely due to hematological toxicity, most commonly severe neutropenia; treatment-related complications were not reported for two of the deceased infants. Long-term neurodevelopmental outcomes were reported as normal in 5 of 13 infants (38.5%), while moderate or severe impairment was described in another five (38.5%). Outcome data were unavailable for the three infants who died (23%).

Clinical features prompting antiviral therapy are summarized in Table 2.

Laboratory abnormalities were the most frequent findings (92.3%), particularly thrombocytopenia (11/13) and leukopenia or neutropenia (7/13). Neuroimaging abnormalities and intrauterine growth restriction or small for gestational age were each reported in 53.8% of cases, although these findings are quite common in the preterm population and are not an indication for antiviral therapy. Hepatitis or cholestasis was observed in 46.2%. Notably, several reports lacked complete clinical descriptions, underscoring the heterogeneity and limitations of available data.

To date, no studies have specifically evaluated the risks and benefits of antiviral therapy in neonates born at such early GAs. Current guidelines recommend antiviral treatment for term neonates with severe symptomatic cCMV infection, whereas treatment of those with moderate disease must be individualized; however, their applicability to extremely preterm infants remains uncertain due to the paucity of safety and efficacy data in this population [11]. As a result, treatment decisions are often extrapolated from studies conducted in term infants, despite substantial biological differences.

Extreme prematurity is characterized by global physiological immaturity, including profound alterations of the immune system [14]. Many immune and organ functions that would normally develop in utero remain immature after preterm birth, leading to immune responses that differ markedly from those of term neonates, particularly with respect to antiviral defense mechanisms [14]. Consequently, both disease course and therapeutic responses in preterm infants may not be directly comparable to those observed in full-term newborns.

Cell-mediated immunity plays a pivotal role in the control of CMV infection [28,29], and in the setting of congenital infection this response is profoundly shaped by fetal and neonatal immune immaturity. Immunoprofiling studies of cord blood have demonstrated that preterm infants exhibit reduced frequencies of key innate and adaptive immune cell populations, together with lower concentrations of pro-inflammatory cytokines such as interleukin (IL)-1β, IL-6, and IL-17A, reflecting an underdeveloped pro-inflammatory response [30]. Experimental studies have further shown that, although Toll-like receptors involved in viral recognition are expressed in preterm neonates, their activation induces a quantitatively and qualitatively attenuated cytokine response—particularly with respect to type I interferon production, which is crucial for early viral control and effective priming of adaptive immunity [28].

Within this immunological context, the virological course observed in our patient is informative. Initiation of GC therapy was associated with a rapid reduction in plasma viremia, suggesting that pharmacological suppression of viral replication may partially compensate for ineffective innate immune control. The subsequent increase in viral load observed 10–20 days after discontinuation of GC may be related to the possible unpredictable absorption of VGC at the beginning of the oral administration or may reflect incomplete immunological containment, attributable to persistent innate immune immaturity and prolonged antigen exposure. Although virus-specific adaptive responses may be activated, they may remain functionally inadequate. Indeed, previous studies have shown that neonates with cCMV exhibit expanded CMV-specific CD4^+^ and CD8^+^ T-cell populations with features of functional exhaustion, including reduced polyfunctionality and increased expression of inhibitory receptors such as PD-1, consistent with impaired viral control under sustained antigenic stimulation [29].

The subsequent decline and clearance of viremia following the introduction of VGC suggest that prolonged suppression of viral replication may reduce antigenic burden and facilitate partial immune reconstitution. The modest and transient rise in viremia observed 30–40 days after discontinuation of VGC is well documented also in term infants [4] and is more consistent with gradual, incomplete restoration of virus-specific immune control than with clinically relevant viral reactivation. In extremely preterm infants, the interaction between attenuated interferon-mediated innate responses, a reduced pro-inflammatory milieu, and an adaptive immune response prone to exhaustion may therefore explain the observed virological dynamics and supports the rationale for early and prolonged antiviral therapy aimed not only at viral suppression but also at indirectly supporting immune maturation.

Following GC initiation, a transient increase in urinary CMV DNA was observed. Although the initial low viral load detected in urine on DOL 3 might be related to a recent fetal infection, this phenomenon has been previously described and is likely attributable to ongoing viral excretion or elimination of residual viral DNA through the urinary tract while systemic replication is already controlled [31]. In addition, sudden interruption of antiviral therapy after 2–3 months of negative viremia and viruria, and consequent lack of immune stimulation by CMV, may be followed by rebound effects due to CMV reactivation. However, polymerase chain reaction detects both infectious virions and non-replicating viral DNA, elevated urinary CMV DNA does not necessarily indicate active viral replication. In light of controlled plasma viremia and clinical stability, urinary CMV monitoring was discontinued in favour of parameters more reflective of active disease and treatment response.

Several studies have demonstrated improved neurodevelopmental outcomes in neonates treated with GC or VGC for cCMV infection [32,33]. In our case, neurodevelopment was normal during follow-up despite early findings of periventricular hyperechogenicity and mild hypotonia—features frequently observed in extremely preterm infants and not necessarily indicative of CMV-related pathology. Given that extreme prematurity itself is a well-established risk factor for adverse neurodevelopmental outcomes [34], the favourable neurological evolution observed in our patient is particularly notable.

The literature highlights substantial heterogeneity in the definition of “symptomatic” cCMV infection, especially with respect to mild or subclinical manifestations, complicating identification of neonates who may benefit from treatment [4]. In reported cases, including ours, neuroimaging abnormalities and laboratory alterations were the most common indications for antiviral therapy. Current guidelines prioritize neurological involvement and hearing impairment as primary criteria for treatment initiation [11,13,35]; however, early identification of these features is challenging in extremely preterm infants.

Laboratory abnormalities may also be treatment-related rather than directly attributable to viral disease, further complicating clinical decision-making. This lack of consensus regarding symptom severity suggests that additional contextual factors—such as extreme prematurity itself—may warrant consideration when evaluating the need for antiviral therapy, particularly if treatment can be shown to be safe and effective in this vulnerable population.

Hematological toxicity remains a major safety concern in the antiviral treatment of cCMV, especially in extremely preterm infants. Both GC and VGC are associated with a high incidence of neutropenia [36,37,38], primarily due to inhibition of viral DNA polymerase and off-target suppression of DNA synthesis in rapidly dividing bone marrow progenitor cells [39,40]. In extremely preterm neonates, this risk is amplified by hematopoietic immaturity, limited bone marrow reserve, reduced granulocytic compensatory capacity, and immature pharmacokinetics that may lead to increased systemic drug exposure even at standard dosages [37,41]. Consequently, neutropenia represents a clinically relevant complication in this population, with potential implications for secondary infections and treatment interruption. In our case, antiviral therapy was maintained without dose modification, as the absolute neutrophil count remained above the recommended discontinuation threshold of 500/mm^3^ [36,37,38]. These findings reinforce the need for individualized treatment decisions, meticulous hematological monitoring, and continuous reassessment of the risk–benefit balance.

A further limitation in managing cCMV infection in extremely preterm infants is the lack of robust pharmacokinetic data to guide dosing. A limitation in our case is that we did not evaluate GCV and VGC plasma concentrations. Our review highlights substantial variability in administered dosages across reported cases. A recent pharmacokinetic study in neonates born before 32 weeks’ gestation represents an important first step toward defining appropriate GC dosing, suggesting that an initial dose of 5 mg/kg every 12 h may be suitable while emphasizing the need for further investigation [41]. Prenatal antiviral therapy with valacyclovir has been shown to reduce vertical transmission and may modulate neonatal disease severity, with a relatively favourable safety profile [42]. More recently, observational data have explored in utero treatment with VGC for symptomatic fetal CMV infection [43]; however, small sample sizes and limited follow-up preclude definitive conclusions regarding long-term safety and efficacy. In our cohort, only the mother of the reported case received prenatal antiviral therapy, limiting assessment of its impact on neonatal outcomes.

In this case, the mother had a reported second-trimester infection and started treatment at 21 weeks of gestation, approximately five weeks before delivery. While this may have influenced the positive outcome of the child, this cannot be confirmed based on a single clinical case, and no other specific information is available regarding maternal viremia. Further studies are needed to evaluate this outcome. Additionally, the vaginal-rectal swab was not tested for the virus. Inhaling viruses during labor can lead to CMV pneumonia, but this cannot be confirmed based on this clinical case.

Collectively, these gaps underscore the urgent need for dedicated studies to define safe and effective antiviral strategies for extremely preterm infants with cCMV infection and to explore the potential role of alternative antiviral agents in this high-risk population.

## 4. Conclusions

Our case report and systematic literature review suggest that antiviral therapy for cCMV infection with GCV and VGC in premature neonates is feasible and safe with careful monitoring, and appears to provide benefits. Nevertheless, well-designed studies that include pharmacokinetics and pharmacodynamics, virologic monitoring, and long term outcomes of development, vision and hearing are urgently needed.

## Figures and Tables

**Table 1:** General data and antiviral treatment and complications data of the case reports of the literature review.

Author	GA BW Sex	Diagnosis	GC/VGC	Start of Treatment (DOL)	Duration of Therapy	Early Cessation of Therapy	Complications of Therapy	Mortality	Long-Term NDV
Sampath 2005 [18]	29 wks 695 g Male	Positive urine culture in the 1 wk of life	GC 12 mg/kg/day	14	exitus	/	ND	25 DOL	Not available
Müller 2008 [19]	28 wks 430 g Male	Positive CMV plasma and urine virus load at 4 DOL	GC 5 mg/kg twice a day VGC 5 mg/kg twice a day	4	GC 35 days VGC 6 weeks	No	ND	No	Mild delay of 4 weeks at Griffith’s test (corrected age of 3 and 6 months)
Koklu 2009 [20]	30 wks 840 g Male	Positive plasma PCR-CMV	GC, doses ND	ND	exitus	/	ND	21 DOL	Not available
Griesmaier 2010 [21]	27 wks 740 g Sex ND	Positive urine PCR-CMV, 5 DOL	GC 7.5 mg/kg/day	7	2 wks	Yes	liver toxicity and neutropenia	No	At 4 months corrected age: extensive truncal hypotonia accompanied by dystonia and tendencies towards hyperextension without asymmetry. Poor repertoire of general movement, retarded mental development. At 1 year corrected age (Bayley Scales-II): PDI < 50, MDI 80.
	27 wks 730 g Sex ND	Positive urine PCR-CMV, 5 DOL	GC 7.5 mg/kg/day	7	2 wks	Yes	liver toxicity and neutropenia	No	Delayed mental development, increased muscle tone with dystonic and hyperextensive compensation and strabismus convergens. Chaotic patterns of general movements. Bayley scale-II not available.
Berardi 2018 [22]	31 wks 1490 g Female	Positive urine CMV-DNA, 4 DOL	GC 6 mg/kg twice a day VGC 6 mg/kg twice a day	GC: 6 VGC: 53	GC: ND VGC: 6 months	Yes, GC	Neutropenia (330/mm^3^)	No	At age 1 year, neurodevelopmental follow-up was within a normal range
Morimoto 2020 [23]	26 wks 1190 g Male	CMV-DNA in blood and urine at DOL28. CMV-DNA in the dried umbilical cord.	GC 12 mg/kg/day	34	6 wks	No	Neutropenia (832/μL)	No	Any neurodevelopmental sequelae noted during the 5-year follow-up
Kanda 2021 [24]	29 wks 1253 g Female	Positive urine CMV-DNA at 7 and 15 DOL	VGC 16 mg/kg twice a day	26	6 months	No	No	No	At 9 months development was age appropriate
Piché-Renaud 2023 [25]	27 wks 1100 g Male	Positive urine CMV-DNA at 4 DOL. Positive blood PCR-CMV	GC 3 mg/kg VGC: 12 mg twice a day, at discharge 16 mg/kg twice a day	GC: 4 VGC: 33	GC: 3 wks VGC: 6 months	No	No	No	At 18 months of corrected age, Bayley-III scores 80, 74, and 79 on cognitive, language, and motor aspects, respectively.
	27 wks 980 g Male	Positive urine CMV-DNA at 4 DOL. Positive blood PCR-CMV	GC 3 mg/kg VGC: 16 mg/kg twice a day	GC: 5 VGC: 59	GC: 54 days VGC: 6 months	Yes, GC. restarted at 16 DOL (5.5 mcg/kg/dose) with G-CSF.	Profound thrombocytopenia (25 × 10^9^/L) and neutropenia (1 × 10^9^/L).	No	
Okahashi 2024 [26]	28 wks 1042 g Female	Positive urine CMV-DNA at 10 DOL	VGC 32 mg/kg/day	67 (38 wks of GA)	26 wks	Yes	Severe neutropenia (<500/mm^3^)	No	Developmental quotient was 104 measured with Kyoto Scale of Psychological Development 2001. The modified checklist for autism in toddlers was negative, indicating no autistic tendencies.
Manalac 2024 [27]	24 wks 355 g Female	CMV DNA PCR not specified if in urine or blood	GC: 6 mg/kg twice a day	Not specified	16 days	No	Thrombocytopenia	32 DOL	Not available
Our report	26 wks 930 g Male	Positive urine CMV-DNA, 3 DOL	GC 6 mg/kg/die VGC 16 mg/kg twice a day	GC: 16 VGC: 93	GC 6 wks VGC 6 months	No	Anemia	No	Adequate neurological development in all the Baylye-III scales

Table legend. GA (Gestational age); BW (Birth weight); DOL (day of life); GV (Ganciclovir); VGC (Valganciclovir); NDV (Neurodevelopment); wks (weeks); g (grams); ND (Not declared); CMV (Cytomegalovirus); PCR (Polymerase Chain Reaction); PDI (psychomotor developmental index); MDI (mental developmental index); G-CSF (granulocyte-colony stimulating factor).

**Table 2:** Symptoms of congenital cytomegalovirus infection in the case reports of the literature review.

Author		Symptoms											
		Abnormal Neuroimaging	Microcephaly	Ocular Impairment	Hearing Impairment	Neurological Abnormal Signs	Abnormal CSF	Abnormal Hematologic Findings	Pneumonitis	Hepatosplenomegaly	IURG or SGA	Hepatitis/ Cholestasis	Other Morbidity
Sampath 2005 [18]		• periventricular calcifications	•	•	•	•	•	• Anaemia, thrombocytopenia, neutropenia	•	•	• IUGR	•	cardiomegaly pericardial effusion, hydrops
Müller 2008 [19]		•	•	•	• (at 6 months)	•	•	• Thrombocytopenia, neutropenia	•	•	• SGA	•	/
Koklu 2009 [20]		•	•	•	•	•	•	• Thrombocytopenia, neutropenia	•	•	• IUGR and SGA	•	pulmonary hypertension, early form of CLD, bilateral pneumothoraces
Griesmaier 2010 [21]		• US: IVH II grade; cystic lesions; calcifications	•	• ROP	•	• seizure	•	• Thrombocytopenia, pathologic Coagulation	•	•	• SGA	•	TTTS (donor), CLD, abdominal wall defect operated on the I DOL
		• US and MRI: bilateral subependymal hemorrhage; cystic lesions; calcifications; cerebellar hypoplasia	•	• ROP	•	• seizure	•	• Thrombocytopenia, pathologic Coagulation	•	•	• SGA	•	TTTS (recipient), CLD
Berardi 2018 [22]		•	•	•	•	•	•	• Thrombocytopenia	•	•	•	•	sepsis-like symptoms with sterile cultures, Stage 2 NEC and non-massive intestinal bleeding.
Morimoto 2020 [23]		•	•	•	•	•	•	•	•	•	•	• elevated hepatic transaminases	ligation of PDA, pneumoperitoneum
Kanda 2021 [24]		•	•	•	•	•	•	• Thrombocytopenia	•	•	•	•	Pulmonary hypertension, *Staphylococcus* epidermidis-induced-CRBSI
Piché-Renaud 2023 [25]		•	•	•	•	•	•	• Leukopenia, thrombocytopenia	•	•	•	•	TTTS, oligohydramnios
		• Hyperechogeniity of the choroid plexus and a discrete white matter bilateral hyperechogenicity	•	•	•	•	•	• Leukopenia, thrombocytopenia	•	•	• IUGR	•	TTTS, polyhydramnios. hypertrophic Cardiomyopathy, grade II NEC
Okahashi 2024 [26]		• Ventricular dilatation	•	• chorioretinitis	•	•	•	• Thrombocytopenia	•	•	•	•	/
Manalac 2024 [27]		• Congenital abnormality of the midline structures, ventriculomegaly, hyperechoic periventricular foci bilaterally, agenesis of corpus callous, calcifications, IVH grade 2, lissencephaly	•	•	•	• seizure	•	• Leukopenia, anemia, thrombocytopenia	•	•	• IUGR	•	/
Our report		• US: hyper-echogenicity of the periventricular brain parenchyma MRI: blurred areas of altered signal in the periventricular white matter	•	•	•	• Mild hypotone	•	• Anemia, neutropenia	•	•	•	•	Late-onset sepsis
%	• Yes	53.8	23.0	23.0	7.7	30.8	7.7	92.3	23.1	7.7	53.8	46.2	
	• NO	30.8	38.5	30.8	53.8	0	7.7	7.7	61.5	23.1	46.2	30.8	
	• ND	15.4	38.5	46.2	38.5	69.2	84.6	0	15.4	69.2	0	23.0	

Table legend. CSF (cerebrospinal fluid); IUGR (intrauterine growth restriction); SGA (small for gestational age); • (Yes); • (No); • (Not declared, ND); CLD (Chronic lung disease); US (ultrasonography); MRI (magnet resonance imaging); TTTS (twin-to-twin transfusion syndrome); IVH (intraventricular hemorrhage); ROP (Retinopathy of prematurity); DOL (day of life); NEC (Necrotizing Enterocolitis); PDA (Patent ductus arteriosus); CRBSI (catheter-related blood stream infection).

## Data Availability

All the available data are included in the manuscript.
